# Effects of Type I Diabetes Mellitus and Masticatory Loading on Mandibular Growth in Growing Rats: A Longitudinal CBCT Study

**DOI:** 10.3390/biology15120979

**Published:** 2026-06-22

**Authors:** Nearchos Panayi, Ismene A. Dontas, Efstathios Chronopoulos, Georgios Kanavakis, Ioannis A. Tsolakis, Georgia Kotantoula, Konstantina Eleni Alexiou, Zafeiroula Yfanti, Orestis Koutras, Apostolos I. Tsolakis

**Affiliations:** 1 Medical School, National and Kapodistrian University of Athens, 10679 Athens, Greece; idontas@med.uoa.gr (I.A.D.); echronop@med.uoa.gr (E.C.); 2 Dental School, European University Cyprus, 2404 Nicosia, Cyprus; o.koutras@external.euc.ac.cy; 3 Dental School, National and Kapodistrian University of Athens, 10679 Athens, Greece; gkanavak@dent.uoa.gr (G.K.); geokot@dent.uoa.gr (G.K.); kalexiou@dent.uoa.gr (K.E.A.); zafeiroula86@gmail.com (Z.Y.); 4 Dental School, Case Western Reserve University, Cleveland, OH 44106, USA; ioannistsolakis@dent.auth.gr; 5 Dental School, Aristotle University of Thessaloniki, 54124 Thessaloniki, Greece

**Keywords:** Type I diabetes mellitus, masticatory loading, Wistar rats, mandibular growth

## Abstract

Background: Craniofacial growth is regulated by a complex interaction of genetic, functional, and systemic metabolic factors. Mechanical loading generated during mastication plays a fundamental role in mandibular development through bone modeling and remodeling mechanisms. In contrast, Type I diabetes mellitus is associated with impaired bone metabolism, which may compromise skeletal growth. Although the independent effects of functional loading and metabolic disorders on bone tissue have been widely investigated, their combined influence on mandibular development remains insufficiently understood. Objective: This study primarily aimed to evaluate the effect of Type I diabetes mellitus on mandibular growth in growing rats and, secondarily, to assess the impact of dietary consistency (hard versus soft food) on mandibular development under diabetic and non-diabetic conditions, as well as determine whether diabetes modifies the mandibular adaptive response to increased masticatory loading. Materials and Methods: An experimental animal study was conducted using twenty-four male Wistar rats aged one month. The animals were randomly allocated into four groups according to metabolic status (control or diabetic) and dietary consistency (hard or soft diet). Type I diabetes mellitus was experimentally induced in the relevant groups using streptozotocin. All animals underwent cone beam computed tomography (CBCT) scanning at baseline (Day 1) and at the end of the experimental period (Day 28). Linear measurements were obtained using specialized software following euthanasia. Two-way ANOVA was used to evaluate the effects of diabetes, diet, and their interaction, using appropriate post hoc tests for multiple comparisons. Categorical variables were analyzed using the chi-square test. A *p*-value < 0.05 was considered statistically significant. Results: Longitudinal morphometric analysis demonstrated that Type I diabetes mellitus significantly impaired mandibular growth. Diabetic animals exhibited reduced growth rates and smaller final mandibular dimensions compared with controls. Hard diet intake significantly enhanced mandibular development in control animals, reflecting a strong adaptive response to increased functional loading. However, this osteogenic response was markedly attenuated in diabetic rats. Under soft-diet conditions, differences between control and diabetic groups were diminished, indicating that reduced mechanical stimulation limits adaptive growth responses. Significant interaction effects between diabetes and dietary consistency were observed in several morphometric parameters, particularly those related to mandibular body length, ramus height, and condylar position. Conclusions: Type I diabetes mellitus exerts a detrimental effect on mandibular growth in growing rats and compromises the adaptive response of craniofacial structures to mechanical loading. Although a hard diet functions as a potent osteogenic stimulus, its growth-promoting effect is substantially reduced in the presence of metabolic dysfunction.

## Simple Summary

Healthy jaw growth depends on two key factors working together: good metabolic health and the mechanical stimulus provided by chewing. This study investigated how Type I diabetes mellitus and dietary consistency (hard versus soft food) affect the growth of the lower jaw (mandible) in growing rats. Twenty-four male Wistar rats were divided into four groups—healthy or diabetic, fed either hard or soft food—and monitored for 28 days using three-dimensional cone beam computed tomography (CBCT) scanning at the start and end of the experiment. Rats that remained healthy and ate hard food showed the greatest jaw growth, driven by the strong mechanical forces of chewing. By contrast, diabetic rats showed significantly reduced jaw growth regardless of diet, and their bones were far less responsive to the stimulating effect of chewing hard food. These results show that diabetes seriously disrupts normal jaw development and weakens the bone’s ability to adapt to mechanical loading. The findings highlight the importance of tight blood sugar control and adequate functional stimulation during the growth years and may help clinicians plan orthodontic treatment more effectively for young patients with Type I diabetes mellitus.

## 1. Introduction

Craniofacial skeletal growth has been interpreted through several complementary, and at times overlapping, theoretical frameworks. The genetic theory holds that growth patterns are largely predetermined by inherited biological programming [1], whereas Moss’s functional matrix theory holds that skeletal structures develop in response to the functional demands of the surrounding soft tissue, emphasizing that function drives form [2,3,4,5,6]. The combined genetic and functional theory reconciles both views, holding that genetic factors set the growth potential while functional and environmental influences shape the final craniofacial morphology [7].

The theory of morphogenetic fields posits that craniofacial development unfolds within spatially organized fields, with distinct regions such as the maxilla and mandible governed by coordinated genetic and cellular interactions [8]. The piezoelectric theory, in turn, proposes that mechanical deformation of bone generates electrical potentials that drive osteoblastic and osteoclastic activity [9]. Enlow’s theory of the counterpart principle and remodeling emphasizes coordinated bone deposition and resorption to maintain structural balance during growth [10,11,12].

Enlow further described primary and secondary displacement, whereby craniofacial growth entails both surface remodeling and spatial repositioning of skeletal units [13]. The concept of messenger-mediated control highlights the role of molecular signaling pathways—including growth factors and cytokines—in orchestrating cellular differentiation and skeletal adaptation [14]. Taken together, these frameworks establish craniofacial growth as a dynamic, multifactorial process shaped by genetic programming, functional adaptation, biomechanical loading, and molecular regulation.

Mandibular growth involves coordinated development of the body and ramus, which support the condyle and coronoid process and provide attachment for the masticatory muscles. Experimental evidence has shown that masticatory muscle function significantly influences mandibular morphology and growth patterns [15,16,17]. The role of the condyle remains debated: Brodie considered it a primary growth driver responsible for forward and downward mandibular displacement [17], whereas Björk, using metallic implants, proposed that condylar growth is mainly compensatory [18]. Enlow and Harris emphasized remodeling and displacement principles, including the “V principle,” as key mechanisms in mandibular growth [11], while Gianelly and Moorrees demonstrated through experimental condylectomies that mandibular length can be maintained via multiple growth sites [19]. Sicher described the condylar cartilage as a primary growth center [20], in contrast to Moss and Salentijn, who argued that it functions as a secondary site influenced by functional matrices [2].

### 1.1. Diabetes Mellitus

Diabetes mellitus is a chronic metabolic disorder characterized by persistent hyperglycemia resulting from impaired insulin secretion or action, with rising global prevalence that places increasing demands on healthcare and dental services [21,22]. Among its many systemic consequences, diabetes disrupts bone metabolism by suppressing osteoblast activity, promoting resorption, and impairing healing [22], rendering the skeleton particularly vulnerable to metabolic compromise. Clinically, diabetes is classified into Type I and Type II forms. Type I diabetes mellitus—formerly called insulin-dependent diabetes—accounts for approximately 10% of all cases and arises from absolute insulin deficiency caused by autoimmune β-cell destruction [23]. It is most prevalent in children and adolescents of European descent. The condition is driven primarily by T-lymphocyte-mediated β-cell destruction; although diabetes-associated autoantibodies were initially regarded as the direct effectors of cellular damage, they are now recognized mainly as amplifiers of the immune response, facilitating CD4+ T-cell accumulation and progressive β-cell loss. Therefore, detection of these autoantibodies serves as a reliable early diagnostic marker for Type I diabetes [24].

The skeletal effects of Type I diabetes during early development remain incompletely characterized, largely because ethical and logistical constraints preclude direct experimental manipulation in growing children, postmortem studies of juvenile specimens are extremely rare, and adult skeletal data are confounded by accumulated endogenous and exogenous factors [25,26]. Experimental animal models are therefore indispensable for mechanistic investigation. Rodents are particularly well suited, offering genetic homogeneity, ease of handling, rapid growth, and close physiological parallels with human skeletal biology. Male Wistar rats are especially susceptible to streptozotocin-induced diabetes and are widely used in metabolic and skeletal research [27,28].

Rats, particularly the Wistar strain, have been extensively employed in craniofacial research, and existing literature provides well-documented evidence of mandibular and maxillary development over short experimental periods of approximately one month [29,30,31,32,33]. Diabetes, especially Type I diabetes mellitus, negatively affects mandibular growth by reducing bone formation, impairing trabecular architecture, and altering mineralization [34,35,36]. Diabetic animal models consistently show decreased mandibular dimensions, compromised bone quality, and delayed skeletal maturation [34,36], with diabetes-related oxidative stress, impaired angiogenesis, and dysregulated gene expression further exacerbating these deficits [37,38]. At the systemic level, diabetes is associated with reduced trabecular bone mass, altered cortical structure, vitamin D deficiency, and elevated fracture risk [34,35,36]. Since mandibular development depends on a precise balance of bone formation and resorption, it is particularly susceptible to diabetes-related metabolic disruption.

### 1.2. The Role of Masticatory Loading on Mandibular Growth

Variations in masticatory muscle function play a crucial role in craniofacial growth by transmitting mechanical forces to the underlying bone. Mechanical loading during mastication acts as a biological stimulus that regulates bone modeling and skeletal development [39,40]. Experimental studies have shown that increased masticatory activity, such as that induced by hard diets, enhances osteocyte activity and stimulates the expression of growth factors such as IGF-1, thereby promoting bone formation [41]. Conversely, reduced muscle function or paralysis following botulinum toxin injections has been associated with significant bone loss in the mandibular condyle and alveolar bone [42,43]. Collectively, experimental and clinical evidence confirms that muscle activity is a key regulator of craniofacial skeletal morphology and development [44]. Furthermore, masseter resection or condylectomy has been shown to impair jaw development and generate facial asymmetries [45,46].

Dietary consistency is one of the most potent environmental modulators of masticatory muscle function and, through it, of mandibular development, since the mechanical forces generated during mastication directly regulate bone modeling and remodeling. Kiliaridis and Shyu showed that rats fed a soft diet exhibited reduced tetanic contraction of the masticatory muscles and diminished mandibular strain [47], resulting in restricted mandibular growth—a finding that underscores the sensitivity of skeletal development to muscle-generated loading. Ödman et al. reported a larger mandibular angle in hard-diet animals [47], whereas Hichijo et al. observed both an increased gonial angle and greater ramus height in that group [48]. Watt et al reportred that rats fed with a hard diet exhibited significantly wider, better-developed dental arches, whereas those on a soft diet showed restricted mandibular and maxillary growth [49].

A substantial body of experimental evidence suggests that a soft diet is associated with a vertical growth pattern of the mandible and a reduction in its overall dimensions [29,50,51,52,53]. In addition, several studies have reported reduced condylar size and decreased cartilage thickness, particularly in the anterior region under conditions of diminished masticatory loading [54,55,56,57,58], indicating that condylar growth is especially sensitive to mechanical stimulation.

In a study using rats in which restriction of the lower jaw was performed, Lyros et al. concluded that mechanical alterations alone may induce localized histological structural remodeling of the condyle without systemic biochemical changes [58].

At the cellular level, the reduced muscular activity associated with a soft diet has been shown to affect chondrocyte proliferation, differentiation, and maturation within the condylar cartilage [59]. Yamamoto further demonstrated that a soft diet restricts bone apposition at the midpalatal suture and reduces transverse maxillary expansion in growing rats, indicating that the effects of mechanical underloading extend beyond the mandible [60].

In a cephalometric study using Sprague Dawley rats, Abed et al. (2007) identified significant differences between hard- and soft-diet groups, with soft-diet animals showing generalized reductions in parameters associated with jaw development [61]. Collectively, these findings indicate that dietary consistency can modify facial morphology, often promoting a dolichofacial skeletal pattern under conditions of reduced masticatory function.

Clinically, decreased masticatory activity has been associated with increased lower anterior facial height, reduced upper anterior facial height, and a tendency toward a long-face phenotype, potentially contributing to skeletal Class II patterns according to Angle’s classification [2,62,63,64]. In contrast, consumption of hard food appears to favor a brachyfacial growth pattern, characterized by increased muscular activity, reduced gonial angle, and enhanced mandibular robustness. These observations underscore the decisive role of mechanical loading in craniofacial skeletal adaptation [2,64].

Despite the extensive literature supporting the influence of muscle function on skeletal development, the precise extent to which changes in muscular activity—through variations in dietary consistency—affect overall mandibular morphology remains incompletely understood. It is still unclear whether functional modifications impact the entire mandibular structure uniformly or whether certain anatomical regions are more sensitive to mechanical stimuli [64]. Taken together, these findings highlight the fundamental importance of functional loading in mandibular development and provide the biological framework for investigating how metabolic disturbances—such as Type I diabetes mellitus—may interact with mechanical factors to influence craniofacial growth.

For these reasons, an experimental study was designed using Wistar rats to primarily investigate the effect of Type I diabetes mellitus on mandibular growth and, secondarily, to evaluate the influence of different masticatory loading conditions on mandibular development. The primary null hypothesis was that no significant differences exist in mandibular growth between diabetic and control animals. The secondary null hypothesis was that dietary consistency, representing high and low masticatory loading, does not affect mandibular growth.

## 2. Materials and Methods

### 2.1. Ethical Approval

The experimental protocol of the study was approved by the Veterinary Directorate of the Attica Region (Protocol No. 725485/29-09-2020; Registration Code EL 25 BIO exp 018). The approval complied with the requirements of Greek National Legislation (Presidential Decree 56/2013) as well as Directive 2010/63/EU of the European Parliament and of the Council (276/33/20.10.2010) on the protection of animals used for scientific purposes.

### 2.2. Experimental Animals

Twenty-four male Wistar rats (one month old) were used. Animals were housed under controlled environmental conditions (temperature 20–22 °C, humidity 55–66%, 12-h light/dark cycle) and had free access to food and water. Animals were monitored regularly for signs of distress or illness throughout the experimental period. No unexpected adverse events occurred, and all procedures were conducted in accordance with approved ethical guidelines. A study protocol including the experimental design and statistical analysis plan was prepared prior to the initiation of the study. A post hoc power analysis (G*Power 3.1) for a two-way ANOVA with α = 0.05 confirmed adequate statistical power (1 − β ≥ 0.80) for the primary outcome measures, given the large effect sizes observed in this and prior studies. No animals were excluded from the analysis, and no missing data occurred.

### 2.3. Experimental Groups

**Figure 1:**
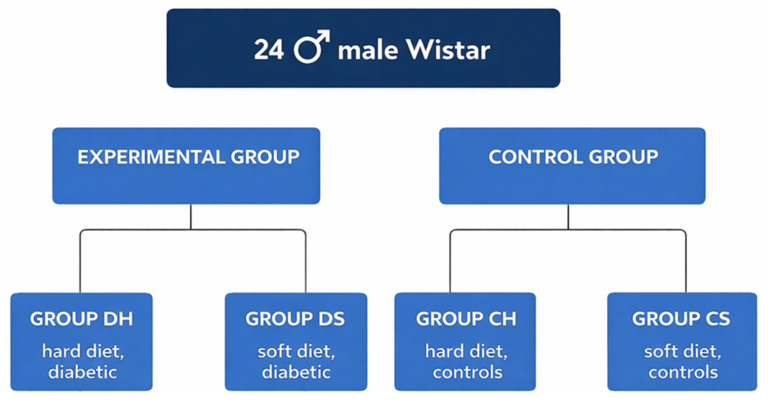
Experimental and control groups.

Rats were randomly allocated into four groups (n = 6 per group) using a computer-generated random number sequence. Group allocation was performed by a researcher not involved in data collection or outcome assessment, providing allocation concealment (Figure 1).

1. Control—Hard diet (CH);
2. Control—Soft diet (CS);
3. Diabetic—Hard diet (DH);
4. Diabetic—Soft diet (DS).

Hard diet: standard laboratory pellets requiring intensive mastication.

Soft diet: pulverized pellets mixed with water to reduce mechanical resistance.

On the 30th day of life (first day of the experiment), twelve rats were rendered diabetic through the intraperitoneal administration of streptozotocin (50 mg/kg), whereas the remaining twelve animals served as the control group. All animals were weighed, and on the same day, cone beam computed tomography (CBCT) scans of the skull were obtained using the CBCT unit of the Dental School of Athens, with the animals positioned in a custom-made device described by Tsolakis [32].

Diabetes induction was confirmed at 3 and 6 days after streptozotocin administration by measuring fasting blood glucose levels. Blood samples were obtained from the tail vein and analyzed using a glucometer (Precision Xtra Blood Glucose System, Abbott, Chicago, IL, USA). Animals with blood glucose levels exceeding 360 mg/dL were considered diabetic. Blood glucose levels and body weight were subsequently monitored every three days, and all animals were regularly assessed to ensure their health and welfare throughout the experimental period. Mean fasting blood glucose at Day 28 was 487.3 ± 38.6 mg/dL in the diabetic groups and 98.4 ± 7.2 mg/dL in the control groups, confirming sustained severe hyperglycemia throughout the experimental period. Mean body weight at Day 28 was 256.3 ± 14.8 g in controls and 187.5 ± 12.4 g in diabetic animals, consistent with the catabolic effects of uncontrolled Type 1 diabetes mellitus (T1DM). The experimental period lasted twenty-eight days. To ensure complete immobilization during imaging, the animals were pre-anesthetized using ether. Specifically, two measured volumes of diethyl ether were placed in a glass container, the animal was placed inside, and a waiting period of 2–3 min was allowed until the animal became calm and exhibited slow, stable respiration.

On Day 28 of the experiment (corresponding to 58 days of age in rats, approximately equivalent to 4.3 years in humans), CBCT scanning of the skull was repeated for all animals following euthanasia. Euthanasia was performed via intramuscular administration of ketamine hydrochloride at a dose of 200 mg/kg body weight.

Scans were performed using a standardized CBCT unit with an 8 × 8 cm field of view and 110 kV exposure, voxel size 0.2 mm, and tube current 5 mA. All scans were evaluated by an experienced radiologist. CBCT landmark identification and linear measurements were performed by two calibrated examiners independently, and both examiners were blinded to group allocation during measurement. Intra-examiner and inter-examiner reliability were assessed using the intraclass correlation coefficient (ICC); ICC values exceeded 0.95 for all measurements, indicating excellent reproducibility. Method error was calculated using Dahlberg’s formula and was below 0.3 mm for all parameters.

### 2.4. Measurements

**Figure 2:**
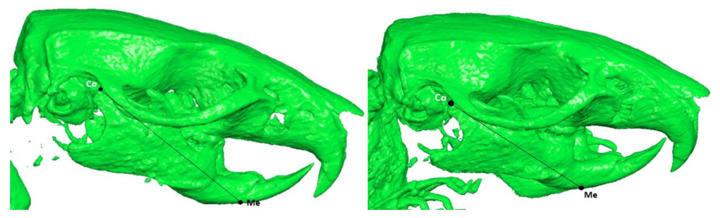
Comparison of the Co–Me measurement between a control rat fed a hard diet (CH) and a diabetic rat fed a hard diet (DH).

Reconstructions of the CBCTs at the beginning and at the end of the experiment were performed and imported into the Viewbox software 4.1.5.5 (Dhal, Kifisia, Athens, Greece). Anatomic points on the 3D images of the rats’ mandibles were used (Table 1, Figure 2). Based on the anatomic points, the following linear measurements using Viewbox were performed:

Go’—Menton (mandibular body length a); Go—Menton (mandibular body length b); Coronoid—Menton; Condylion/Go’—Menton (Condylion height); Condylion—Go’ (Ramus height); Condylion—Menton; Condylion—Id; Condylion—I’ (mandibular length); Incisal—Id; Incisal—I’ (Figure 2).

**Table 1:** Three-dimensional reconstruction and measurements were performed using Viewbox software.

Skeletal Landmarks	Description
Condylion right	The most superior and posterior point of the right condyle
Go right	The most posterior apex of the right gonial process
Go’ right	The most inferior point of the right gonial process
Coronoid right	The apex of the right coronoid process
Menton right	The most inferior point of the right mental process
Id right	The highest point of the alveolar process between the lower incisors in the right anterior region
I’ right	The most anterior edge of the alveolar petal at the curvature of the right lower incisor
Condylion left	The most superior and posterior point of the left condyle
Go left	The most posterior apex of the left gonial process
Go’ left	The most inferior point of the left gonial process
Coronoid left	The apex of the left coronoid process
Menton left	The most inferior point of the left mental process
Id left	The highest point of the alveolar process between the lower incisors in the left anterior region
I’ left	The most anterior edge of the alveolar petal at the curvature of the left lower incisor

### 2.5. Statistical Analysis

All growth analyses were based on change scores (Δ values = Day 28 − Day 1 measurements) and on Day 28 as a finishing time point representing net growth increments over the experimental period. The use of Δ values as the unit of analysis is methodologically appropriate for a two-way ANOVA without repeated measures, as it removes the need to account for baseline covariation and is consistent with published practice in comparable longitudinal rat studies. A two-way analysis of variance (two-way ANOVA) without repeated measures was performed to evaluate the effects of diabetes status (yes/no) and dietary consistency (hard/soft), as well as their interaction. In the absence of a statistically significant interaction, main effects were analyzed independently. Multiple comparisons were performed using the Scheffé post hoc test. When a significant interaction was detected, a combined factor including the four experimental groups (control—hard diet, diabetic—hard diet, control—soft diet, diabetic—soft diet) was created, and one-way ANOVA was applied, followed by Bonferroni-adjusted post hoc comparisons. When assumptions of normality or homogeneity of variance were violated, non-parametric tests (Kruskal–Wallis and Mann–Whitney U tests) were used for group comparisons, and Welch’s ANOVA with Games–Howell post hoc tests was applied for unequal variances. Categorical variables between groups were compared using the chi-square test or Fisher’s exact test, with Bonferroni correction for multiple comparisons.

All statistical analyses were conducted using SPSS software (version 21.0; IBM Corporation, Somers, NY, USA). All tests were two-sided, and a *p*-value < 0.05 was considered statistically significant. Marginally significant differences (0.05 < *p* < 0.10) were also reported. The data supporting the findings of this study are available from the corresponding author upon reasonable request.

## 3. Results

### 3.1. Effect of Diabetes and Masticatory Loading on Mandibular Growth (Table 2, Table 3 and Table 4)

Growth Changes from Day 1 to Day 28 (Dynamic Growth Phase).

The longitudinal analysis from Day 1 to Day 28 demonstrated that both diabetes and dietary consistency significantly influenced mandibular growth. For most linear measurements, including Go’–Menton, Go–Menton, Incisal ld, Incisal l’, Condylion ld, Condylion–Menton, Condylion–Go’, and Coronoid–Menton, a statistically significant interaction between diabetes and diet was observed.

Under hard-diet conditions, control animals exhibited significantly greater increases in mandibular dimensions than diabetic animals, confirming that diabetes impairs the adaptive growth response to increased functional loading. Under soft-diet conditions, inter-group differences were reduced or absent, suggesting that diminished mechanical stimulation limits the detectable metabolic impact on mandibular growth—a pattern particularly evident in parameters such as Go’–Menton and Go–Menton, where low-loading conditions may be insufficient to expose metabolic differences. An exception to this pattern was observed for the Coronoid–Menton measurement under soft-diet conditions, where diabetic animals exhibited a larger increment than controls (17.35 ± 0.48 mm vs. 11.55 ± 1.02 mm, *p* < 0.005). This finding runs counter to the general trend and warrants careful interpretation. The coronoid process is primarily influenced by temporal muscle function rather than condylar growth mechanisms, and its growth trajectory may differ from other mandibular parameters. We hypothesize that under conditions of reduced overall mandibular loading (soft diet), the temporal muscle attachment region may experience relatively preserved or redistributed mechanical forces, potentially explaining the divergent response. However, this finding should be interpreted cautiously, and its biological significance requires confirmation in future studies with molecular markers of muscle–bone interaction. The authors acknowledge this as a data point requiring further investigation and not consistent with a uniform interpretation of diabetes-induced growth attenuation across all mandibular regions.

Hard diet enhanced growth in most parameters across both groups; however, the magnitude of this response was consistently attenuated in diabetic animals, indicating that the anabolic effects of mechanical loading are significantly curtailed under diabetic conditions.

**Table 2:** (**a**,**b**) Differences in linear measurement results for 0–28 days of observation.

Measurement	Diet	Control	Diabetes	*p*-Value Diabetes	Diet-Independent Diabetes	Interaction Diabetes–Diet
(**a**)						
Go–Menton	Soft diet	16.54 ± 1.56	16.07 ± 0.75	1.000	16.30 ± 0.35	F(1, 20) = 272.2 *p* < 0.005
	Hard diet	36.25 ± 0.92	19.46 ± 1.42	*p* < 0.005	27.86 ± 0.35	
	*p*-value diet	*p* < 0.005	*p* < 0.005		*p* < 0.005	
	Diet-independent	26.39 ± 0.35	17.77 ± 0.35	*p* < 0.005		
Intercondylar Distance	Soft diet	1.00 ± 0.22	0.91 ± 0.28	1.000	0.95 ± 0.09	F(1, 20) = 0.74 *p* = 0.400
	Hard diet	1.72 ± 0.23	1.42 ± 0.44	0.619	1.57 ± 0.09	
	*p*-value diet	*p* < 0.005	*p* = 0.056			
	Diet-independent	1.36 ± 0.09	1.16 ± 0.09	*p* = 0.136		
Coronoid– Menton	Soft diet	11.55 ± 1.02	17.35 ± 0.48	*p* < 0.005	14.45 ± 0.43	F(1, 20) = 5.43 *p* = 0.030
	Hard diet	20.82 ± 2.59	23.81 ± 0.84	*p* = 0.124	22.32 ± 0.43	
	*p*-value diet	*p* < 0.005	*p* < 0.005		*p* < 0.005	
	Diet-independent	16.19 ± 0.43	20.58 ± 0.43	*p* < 0.005		
Incisal–Id	Soft diet	19.10 ± 1.28	16.90 ± 0.89	*p* = 0.031	18.00 ± 0.41	F(1, 20) = 99.78 *p* < 0.005
	Hard diet	35.79 ± 2.27	22.10 ± 0.56	*p* < 0.005	28.96 ± 0.41	
	*p*-value diet	*p* < 0.005	*p* < 0.005		*p* < 0.005	
	Diet-independent	27.44 ± 0.41	19.52 ± 0.41	*p* < 0.005		
Condylion–Id	Soft diet	6.79 ± 2.04	12.45 ± 0.83	*p* < 0.005	9.62 ± 0.33	F(1, 20) = 264.61 *p* < 0.005
	Hard diet	23.81 ± 0.44	14.25 ± 0.44	*p* < 0.005	19.03 ± 0.33	
	*p*-value diet	*p* < 0.005	*p* = 0.008		*p* < 0.005	
	Diet-independent	15.30 ± 0.33	13.35 ± 0.33	*p* < 0.005		
(**b**)						
Condylion Go’–Menton	Soft diet	5.18 ± 2.98	8.57 ± 0.47	0.128	6.88 ± 0.47	F(1, 20) = 74.93 *p* < 0.005
	Hard diet	17.89 ± 1.07	9.83 ± 0.47	*p* < 0.005	13.86 ± 0.47	
	*p*-value diet	*p* < 0.005	*p* < 0.005		*p* < 0.005	
	Diet-independent	11.53 ± 0.47	9.20 ± 0.47	*p* < 0.005		
Condylion–Go’	Soft diet	8.96 ± 1.93	11.70 ± 0.76	*p* = 0.008	10.35 ± 0.37	F(1, 20) = 8.64 *p* = 0.008
	Hard diet	20.76 ± 0.85	20.45 ± 1.26	*p* = 1.000	20.61 ± 0.37	
	*p*-value diet	*p* < 0.005	*p* < 0.005		*p* = 0.029	
	Diet-independent	14.86 ± 0.37	16.10 ± 0.37			
Incisal–I’	Soft diet	19.66 ± 1.60	20.73 ± 2.48	*p* = 1.000	20.20 ± 0.54	F(1, 20) = 137.35 *p* < 0.005
	Hard diet	38.21 ± 2.07	21.39 ± 1.00	*p* < 0.005	29.80 ± 0.54	
	*p*-value diet	*p* < 0.005	1.000		*p* < 0.005	
	Diet-independent	28.94 ± 0.54	21.06 ± 0.54	*p* < 0.005		
Go’–Menton	Soft diet	18.18 ± 1.29	17.55 ± 1.10	0.798	17.88 ± 0.56	F(1, 20) = 32.04 *p* < 0.005
	Hard diet	36.58 ± 3.38	27.00 ± 0.83	*p* < 0.005	31.79 ± 0.56	
	*p*-value diet	*p* < 0.005	*p* < 0.005		*p* < 0.005	
	Diet-independent	27.38 ± 0.56	22.27 ± 0.56	*p* < 0.005		
Go–Menton	Soft diet	7.73 ± 1.31	10.77 ± 0.92	*p* < 0.005	9.25 ± 0.36	F(1, 20) = 58.38 *p* < 0.005
	Hard diet	18.54 ± 1.73	13.83 ± 0.78	*p* < 0.005	16.19 ± 0.36	
	*p*-value diet	*p* < 0.005	*p* < 0.005		*p* < 0.005	
	Diet-independent	13.14 ± 0.36	12.30 ± 0.36	*p* = 0.114		

### 3.2. Absolute Measurements at Day 28 (Final Morphology) (Table 3 and Table 4)

Evaluation of absolute mandibular dimensions at Day 28 revealed distinct morphological patterns depending on metabolic status and dietary consistency. Significant interactions between diabetes and diet were observed in most linear parameters.

Control animals generally exhibited larger final mandibular dimensions compared with diabetic animals, particularly under hard-diet conditions. These findings indicate a persistent inhibitory effect of diabetes on mandibular development, which was not fully compensated for by increased masticatory loading.

The hard diet resulted in significantly greater final values in multiple measurements, confirming the long-term osteogenic effect of increased functional loading. However, this anabolic response—reflecting enhanced bone formation—was markedly reduced in diabetic rats. This attenuation suggests impaired mechanotransduction, meaning a diminished capacity of bone tissue to convert mechanical strain into biological signals that activate osteoblast-mediated bone formation. It must be emphasized, however, that mechanotransduction was not directly assessed in the present study; no molecular markers (e.g., sclerostin, osteocalcin, and IGF-1), osteocyte gene expression data, or bone formation markers were measured. The interpretation of impaired mechanotransduction, therefore, represents a biologically plausible hypothesis consistent with the morphometric findings and the existing literature on diabetic bone, rather than a directly demonstrated mechanistic conclusion. Future studies incorporating bone histomorphometry and mechanosensing pathway analysis would be required to substantiate this mechanism.

**Table 3:** (**a**,**b**) Linear measurement results for Day 28 (last day of the experiment).

Measurement	Diet	Control	Diabetes	*p*-Value Diabetes	Diet-Independent Diabetes	Interaction Diabetes–Diet
(**a**)						
Go’–Menton	Soft diet	15.52 ± 0.12	15.65 ± 0.21	0.548	15.58 ± 0.07	F(1, 20) = 61.36 *p* < 0.005
	Hard diet	18.98 ± 0.15	17.62 ± 0.38	*p* < 0.005	18.30 ± 0.07	
	*p*-value diet	*p* < 0.005	*p* < 0.005		*p* < 0.005	
	Diet-independent	17.25 ± 0.07	16.63 ± 0.07	*p* < 0.005		
Intercondylar Distance	Soft diet	18.60 ± 0.11	18.52 ± 0.08	*p* = 1.000	18.56 ± 0.04	F(1, 20) = 16.10 *p* < 0.005
	Hard diet	18.73 ± 0.18	19.08 ± 0.15	*p* < 0.005	18.91 ± 0.04	
	*p*-value diet	*p* = 0.577	*p* < 0.005		*p* < 0.005	
	Diet-independent	18.67 ± 0.04	18.80 ± 0.04	*p* = 0.023		
Coronoid–Menton	Soft diet	16.10 ± 0.10	16.80 ± 0.24	*p* < 0.005	16.45 ± 0.05	F(1, 20) = 164.10 *p* < 0.005
	Hard diet	17.65 ± 0.21	20.28 ± 0.17	*p* < 0.005	18.97 ± 0.05	
	*p*-value diet	*p* < 0.005	*p* < 0.005		*p* < 0.005	
	Diet-independent	16.88 ± 0.05	18.54 ± 0.05	*p* < 0.005		
Incisal–Id	Soft diet	9.67 ± 0.14	9.57 ± 0.12	1.000	9.62 ± 0.04	F(1, 20) = 72.37 *p* < 0.005
	Hard diet	11.13 ± 0.12	10.12 ± 0.15	*p* < 0.005	10.63 ± 0.04	
	*p*-value diet	*p* < 0.005	*p* < 0.005		*p* < 0.005	
	Diet-independent	10.40 ± 0.04	9.84 ± 0.04	*p* < 0.005		
Condylion–Id	Soft diet	21.77 ± 0.41	23.03 ± 0.14	*p* < 0.005	22.40 ± 0.09	F(1, 20) = 149.40 *p* < 0.005
	Hard diet	25.57 ± 0.20	23.92 ± 0.34	*p* < 0.005	24.74 ± 0.09	
	*p*-value diet	*p* < 0.005	*p* < 0.005		*p* < 0.005	
	Diet-independent	23.67 ± 0.09	23.48 ± 0.09	*p* = 0.124		
(**b**)						
Condylion–Menton	Soft diet	19.35 ± 0.45	20.27 ± 0.15	*p* < 0.005	19.81 ± 0.08	F(1, 20) = 64.93 *p* < 0.005
	Hard diet	21.87 ± 0.12	21.03 ± 0.22	*p* < 0.005	21.45 ± 0.08	
	*p*-value diet	*p* < 0.005	*p* < 0.005		*p* < 0.005	
	Diet-independent	20.61 ± 0.08	20.65 ± 0.08	*p* = 0.705		
Condylion Go’–Menton	Soft diet	8.93 ± 0.16	9.52 ± 0.08	*p* < 0.005	9.23 ± 0.04	F(1, 20) = 0.115 *p* = 0.738
	Hard diet	10.18 ± 0.08	10.80 ± 0.14	*p* < 0.005	10.49 ± 0.04	
	*p*-value diet	*p* < 0.005	*p* < 0.005		*p* < 0.005	
	Diet-independent	9.56 ± 0.04	10.16 ± 0.04	*p* < 0.005		
Incisal–I’	Soft diet	6.08 ± 0.15	6.12 ± 0.12	1.000	6.10 ± 0.03	F(1, 20) = 59.65 *p* < 0.005
	Hard diet	7.12 ± 0.10	6.43 ± 0.08	*p* < 0.005	6.78 ± 0.03	
	*p*-value diet	*p* < 0.005	*p* < 0.005		*p* < 0.005	
	Diet-independent	6.60 ± 0.03	6.28 ± 0.03	*p* < 0.005		
Go–Menton	Soft diet	17.77 ± 0.25	17.30 ± 0.34	*p* = 0.047	17.53 ± 0.08	F(1, 20) = 16.86 *p* < 0.005
	Hard diet	20.88 ± 0.26	21.33 ± 0.23	*p* = 0.059	21.10 ± 0.08	
	*p*-value diet	*p* < 0.005	*p* < 0.005		*p* < 0.005	
	Diet-independent	19.33 ± 0.08	19.32 ± 0.08	*p* = 0.941		
Go’–Menton	Soft diet	9.07 ± 0.10	9.60 ± 0.11	*p* < 0.005	9.33 ± 0.03	F(1, 20) = 11.91 *p* < 0.005
	Hard diet	10.23 ± 0.14	10.43 ± 0.12	*p* = 0.050	10.33 ± 0.03	
	*p*-value diet	*p* < 0.005	*p* < 0.005		*p* < 0.005	
	Diet-independent	9.65 ± 0.03	10.02 ± 0.03	*p* < 0.005		

**Table 4:** Comparative outcomes across the four experimental groups.

Index	Control—Hard Diet	Diabetic—Hard Diet	Control—Soft Diet	Diabetic—Soft Diet
Go’–Menton	Greatest increase; highest final values	Reduced increase vs. control—hard	Moderate increase	Lowest increase
Go–Menton	Significant growth enhancement	Attenuated growth response	Moderate growth	Reduced growth
Incisal–Id	Marked increase	Reduced but present increase	Moderate increase	Lower increase
Incisal–I’	Significant elongation	Reduced elongation	Mild elongation	Minimal elongation
Condylion–Id	Strong adaptive growth	Impaired adaptive response	Moderate adaptation	Reduced adaptation
Condylion–Menton	Increased vertical dimension	Reduced vertical increase	Moderate increase	Lower increase
Condylion–Go’	Enhanced ramus development	Blunted response	Moderate development	Reduced development
Coronoid–Menton	Increased ramus- coronoid growth	Attenuated increase Paradoxical: greater increment than CH (23.81 vs. 20.82 mm)	Moderate growth	Paradoxical: greater increment than CS (17.35 vs. 11.55 mm)
Intercondylar Distance	Higher final values	Slightly reduced vs. control—hard	Moderate values	Lowest values

## 4. Discussion

Craniofacial growth is a complex, multidimensional biological process regulated by the interaction of genetic determinants, systemic metabolic status, and functional environmental stimuli. The mandible, as a principal structural component of the craniofacial complex, undergoes continuous modeling and remodeling throughout childhood and adolescence, with adaptive remodeling continuing into adulthood [10]. Its development depends not only on intrinsic genetic programming but also on extrinsic functional loading and systemic hormonal balance. Among systemic disorders capable of disrupting skeletal growth, Type I diabetes mellitus (T1DM) represents a profound metabolic condition characterized by insulin deficiency and chronic hyperglycemia, both of which significantly affect bone metabolism [22,23]. Simultaneously, masticatory loading functions as a major regulator of mandibular growth, influencing both bone quantity and morphology [41,42,43,44,45,46,47,48,49,50,51,52,53,54,55,56]. The present study investigated the independent and combined effects of metabolic dysregulation and altered mechanical loading on mandibular development in growing Wistar rats.

The influence of masticatory muscle activity on craniofacial morphology has been well established. Experimental interventions modifying muscle function—such as denervation, myotomy, occlusal alteration, functional appliances, and dietary consistency modification—have consistently demonstrated that skeletal structures adapt to altered mechanical demands [2,41,42,43,44,45,46,47,48,49,50,51,52,53,54,55,56,65,66,67]. Hard diets increase masticatory effort and generate greater strain magnitude and frequency within the mandible and temporomandibular joint, whereas soft diets reduce mechanical stimulation. Bone tissue responds to such mechanical forces through mechanotransduction, a process by which osteocytes detect strain and translate it into biochemical signals regulating osteoblast differentiation and bone deposition [64,68]. This adaptive mechanism ensures that mandibular morphology reflects its biomechanical environment, which is consistent with Wolff’s law and contemporary mechanobiological theory [65].

In contrast, Type I diabetes mellitus creates a metabolically unfavorable environment for skeletal growth. Streptozotocin (STZ)-induced experimental diabetes is the most widely used model of Type I diabetes mellitus in rats [34,35,36,69,70]. Chronic hyperglycemia impairs osteoblast proliferation and differentiation, enhances osteoclast activity, and reduces bone formation rates [71,72], leading to decreased bone mass and compromised microarchitecture. Craniofacial structures are similarly susceptible; studies on diabetic Wistar rats have consistently reported reduced mandibular dimensions, decreased bone volume, and impaired structural integrity [34,35,36,70]. These changes are mechanistically linked to insulin deficiency and downregulation of insulin-like growth factor-1 (IGF-1), a critical anabolic mediator of osteoblast survival, collagen synthesis, and matrix mineralization [71], the disruption of which substantially curtails the skeletal growth potential of diabetic animals.

The present findings confirm that both metabolic status and dietary consistency significantly influence mandibular growth. Hard diet consumption consistently promoted greater increases in mandibular linear measurements compared with soft diet consumption, reinforcing the anabolic effect of mechanical loading. These results are consistent with previous reports demonstrating that increased masticatory demand enhances mandibular length, cortical thickness, and overall bone mass [32,41,44,46,47,51]. Functional stimulation promotes osteocyte activation, increases local growth factor production, and enhances bone formation aligned with mechanical stress vectors [72,73].

However, a central observation of this study was that the osteogenic response to a hard diet was markedly attenuated in diabetic animals. Although diabetic rats exposed to increased mechanical loading exhibited measurable growth, their increments and final mandibular dimensions remained significantly lower than those of healthy controls. This finding indicates that diabetes compromises the skeleton’s adaptive capacity. Bone adaptation to mechanical strain depends on intact osteocyte mechanosensing and downstream signaling pathways regulating osteoblast recruitment [74]. In diabetic conditions, oxidative stress and collagen glycation alter the bone matrix and impair cellular communication [75]. Advanced glycation end products (AGEs) accumulate within collagen fibers, increasing stiffness while reducing matrix quality, which may interfere with strain detection and cellular signaling efficiency [76].

Furthermore, diabetes is associated with elevated reactive oxygen species (ROS) and increased expression of pro-inflammatory cytokines such as IL-6 and TNF-α [77]. These mediators inhibit osteoblast differentiation and promote osteoclastogenesis, shifting bone homeostasis toward resorption. Even in the presence of mechanical stimulation, the biological environment required for optimal anabolic response is compromised. Thus, metabolic dysregulation reduces the effectiveness of mechanotransduction pathways and limits adaptive growth.

The interaction between diabetes and dietary consistency highlights the multifactorial regulation of mandibular development. Reduced mechanical loading (soft diet) resulted in diminished growth across groups, confirming the fundamental role of function in skeletal modeling. Conversely, increased loading (hard diet) significantly enhanced growth in healthy animals but only partially compensated for diabetic impairment. This suggests that mechanosensitivity is not entirely abolished in diabetic bone but operates at a reduced efficiency. The diabetic skeleton retains some capacity to respond to mechanical stimulation, yet its anabolic response is blunted.

These findings align with mechanobiological theory, which proposes that bone mass and architecture are determined by the balance between mechanical demand and biological responsiveness [78]. In healthy animals, increased strain surpasses the modeling threshold, triggering bone formation. In diabetic animals, this threshold may be elevated due to impaired cellular signaling, requiring greater mechanical input to achieve similar anabolic effects [79]. Consequently, even increased masticatory loading may be insufficient to fully restore normal growth trajectories under diabetic conditions.

The complementary analysis of growth increments and final dimensions—capturing dynamic adaptation rates and cumulative outcomes, respectively—consistently showed reduced values in diabetic animals, supporting the concept of sustained metabolic inhibition throughout the experimental period.

Clinically, these findings have important implications. Children and adolescents with Type I diabetes may experience altered craniofacial growth patterns, potentially affecting mandibular growth, occlusal relationships, and facial proportions. Orthodontic diagnosis and treatment planning in diabetic patients should consider possible differences in growth velocity and skeletal responsiveness. Moreover, the results emphasize the importance of glycemic control during growth phases. Optimal metabolic regulation may preserve osteoblastic function and maintain mechanosensitivity. More specifically, the timing of orthodontic intervention in pediatric diabetic patients may need to be adapted to account for reduced growth velocity and attenuated responsiveness to functional appliances. Functional therapies that rely on condylar growth stimulation (e.g., activators, twin blocks) may be less effective in poorly controlled diabetic adolescents, potentially requiring longer treatment durations or more intensive force delivery. Clinicians should consider integration with the patient’s endocrinology team to optimize glycemic status prior to and during active growth modification phases. Additionally, the finding that even increased functional loading only partially compensates for metabolic impairment underscores the primacy of metabolic management over purely mechanical intervention strategies in this patient population.

The growth-promoting effect of hard diets observed in healthy animals has broader relevance: the progressive shift towards softer diets in modern populations may reduce habitual masticatory demand and thereby attenuate craniofacial skeletal stimulation—a biologically plausible concern, though extrapolation from rodent to human physiology must be made with caution.

Several limitations warrant acknowledgement. The 28-day observation window captures a discrete developmental phase and may not reflect longer-term adaptation or compensatory growth. The STZ model faithfully reproduces key features of Type I diabetes mellitus but does not replicate the full metabolic heterogeneity of the human condition, and inherent interspecies differences limit direct clinical extrapolation. Nonetheless, because ethical constraints preclude comparable experimental manipulation in growing children, animal models remain indispensable for the mechanistic investigation of this kind. No insulin treatment was administered, resulting in severe, uncontrolled hyperglycemia that may represent an extreme metabolic state not fully reflective of managed clinical Type 1 diabetes mellitus (T1DM). The absence of direct measures of mechanotransduction (e.g., bone histomorphometry, osteocyte markers, and molecular signaling assays) means that the proposed mechanistic interpretation remains inferential. Furthermore, the study did not include dietary intake monitoring, so differences in caloric consumption between hard- and soft-diet groups cannot be entirely excluded as a contributing factor. Finally, the study examined only male rats; hence, sex-related differences in the interaction between diabetes and masticatory loading on craniofacial growth remain to be investigated.

Future studies employing longer observation periods, graded glycemic control, and molecular profiling of mechanotransduction pathways would further clarify the metabolic–mechanical interaction described here, and investigations into strategies for restoring mechanosensitivity in diabetic bone may hold translational therapeutic value.

In conclusion, mandibular growth is governed by a dynamic interplay between systemic metabolic status and mechanical loading. Type I diabetes mellitus substantially impairs skeletal growth and attenuates adaptive responsiveness to masticatory strain, while increased functional loading exerts a strong anabolic influence that partially—but not fully—compensates for metabolic deficits. These findings deepen the understanding of craniofacial growth regulation and highlight the clinical imperative to integrate glycemic management and functional stimulation when planning orthodontic treatment in growing diabetic patients.

## 5. Conclusions

In growing rats, mandibular development is governed by the interplay between systemic metabolic health and mechanical stimulation from mastication. Type I diabetes mellitus impairs growth velocity, reduces final mandibular dimensions, and blunts adaptive responsiveness to functional loading. Although a hard diet acts as a potent osteogenic stimulus, its growth-promoting effect is substantially diminished under diabetic conditions. These findings underscore the importance of both glycemic regulation and adequate functional stimulation for normal craniofacial development, with potential implications for the prevention and management of skeletal growth disturbances in young patients with Type I diabetes mellitus.

## Data Availability

The data supporting the findings of this study are available from the corresponding authors upon reasonable request.
